# Influence of Maternal Inulin-Type Prebiotic Intervention on Glucose Metabolism and Gut Microbiota in the Offspring of C57BL Mice

**DOI:** 10.3389/fendo.2019.00675

**Published:** 2019-10-01

**Authors:** Qian Zhang, Xinhua Xiao, Jia Zheng, Ming Li, Miao Yu, Fan Ping, Tong Wang, Xiaojing Wang

**Affiliations:** Key Laboratory of Endocrinology, Ministry of Health, Department of Endocrinology, Peking Union Medical College Hospital, Peking Union Medical College, Chinese Academy of Medical Sciences, Beijing, China

**Keywords:** maternal, offspring, inulin, glucose metabolism, gut microbiota

## Abstract

**Scope:** Maternal obesity leads to glucose intolerance in the offspring. Changes in the gut microbiota are being increasingly implicated in the pathogenesis of diabetes. We hypothesized that inulin intervention during gestation and lactation improves glucose metabolism disorders in mouse offspring from high-fat diet (HD)-fed dams.

**Procedures:** Female C57BL mice were fed a control diet or HD for 4 weeks before mating. After mating, pregnant mice were randomly divided into three groups through gestation and lactation: control diet (CD) group, HD group, and HD treated with inulin (HD-inulin) group. At weaning, glucose metabolic status was assessed. Gut microbial DNA from offspring cecal contents was isolated and processed for metagenomic shotgun sequencing, and taxonomic and functional profiling were performed.

**Results:** Offspring from dams in the HD-inulin groups demonstrated reduced fasting blood glucose, decreased blood glucose area under the curve during the oral glucose tolerance test, and reduced fasting serum insulin and HOMA-IR compared to offspring from dams in the HD group. Nineteen differentially abundant bacterial species were identified between the HD-inulin and HD groups. The HD-inulin group displayed significantly greater abundances of *Bacteroides_acidifaciens, Eubacterium_sp_CAG_786, Clostridium_sp_CAG_343*, and *Bifidobacterium_breve* species and lower abundances of *Oscillibacter_sp_1_3, Ruminococcus_gnavus_CAG_126*, and *Bacteroides_massiliensis* species. Differentially abundant bacterial species among the three groups were involved in 38 metabolic pathways, including several glucose and lipid metabolism pathways.

**Conclusion:** Our results show that early inulin intervention in HD-fed mouse dams moderates offspring glucose metabolism and gut dysbiosis.

## Introduction

Nutrition status during the intrauterine period has been reported to lead to the programming of metabolic disorders in the offspring throughout their whole life span (1). Maternal overnutrition can program an increased risk of diabetes in rodent offspring (2, 3). Improving maternal metabolism may help curb the burden of metabolic disease in offspring (4). However, the mechanism through which beneficial outcomes are programmed in offspring remains to be elucidated. Increasing evidence has shown that the gut microbiota greatly affects host metabolism (5–7). Type 2 diabetes patients have dysbiotic microbiota, which plays a central role in the process of diabetes and provides unique markers for its diagnosis (8). Transplantation of “diabetic microbiota” to germ-free mice results in insulin resistance (9). Moreover, the maternal gut microbiota has an important contribution to the colonization of the offspring gut (10) and impacts long-term metabolic health (11). These studies suggest that moderation of the maternal gut microbiota may be a potential target for the prevention of diabetes in offspring.

Dietary inulin-type prebiotic treatment represents a promising strategy for altering the gut microbiota and affecting host metabolism and physiology (12, 13). As one type of prebiotic, inulin is extracted from chicory roots. Inulin cannot be hydrolyzed by digestive enzymes in the human small intestine but is fermented by probiotics (12). Inulin intake has been proven to reduce blood glucose and moderate insulin resistance in diabetic rodent models (14, 15). In clinical trials, inulin supplementation was found to moderate glycemic status in diabetic patients (16). Furthermore, in high-fat diet (HD)-induced diabetic rats, inulin had a beneficial impact on gut microbiota profiles (17).

We hypothesized that inulin intervention during gestation and lactation improves glucose metabolism disorder in offspring from HD-fed dams. To identify this alteration in gut microbiota, we used a metagenomic shotgun sequencing approach to analyze the gut microbiota of offspring from inulin-supplemented HD-fed dams. In this study, we used metagenomic shotgun sequencing to sequence the whole set of genes present in the gut microbiome. This sequencing information can provide the relative abundance of genes not only in functional pathways but also at all taxonomical levels (18). The aim of this project was to provide a comprehensive understanding of the gut microbial mechanism in offspring of HD-fed dams receiving early inulin intervention.

## Materials and Methods

### Animal Treatments and Diets

**Figure 1:**
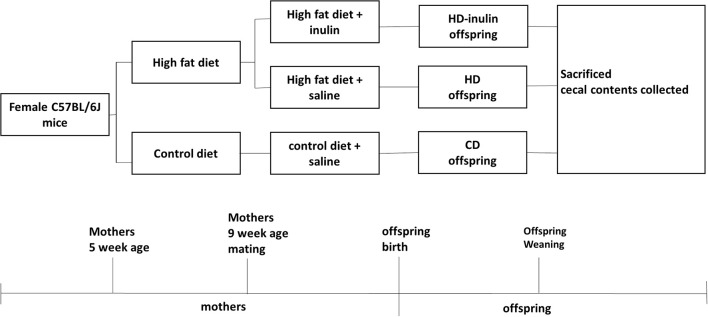
Timeline of animal protocol for different groups. CD, control diet; HD, high fat diet; HD-inulin, high fat diet with inulin supplement.

All animal experimental protocols were approved by the Animal Care Committee of Peking Union Medical Hospital (Permit Number: MC-07-6004). Five-week-old female C57BL6/J mice (body weight 13.03 ± 0.79 g) were given *ad libitum* access to control (kcal %: 10% fat, 20% protein, and 70% carbohydrate; 3.85 kcal/gm, *n* = 8) or high-fat diets (kcal %: 45% fat, 20% protein, and 35% carbohydrate; 4.73 kcal/gm; *n* = 16) for 4 weeks. At 9 weeks of age, females were bred with control male mice fed with a control diet. The vaginal plug was checked to confirm pregnancy. During the gestation and lactation period, female mice fed a control diet before gestation remained on a control diet (CD); female mice fed a high-fat diet before gestation went on either a high-fat diet (HD) only or a high-fat diet with 10% wt/wt inulin supplementation (HD-inulin, Vilof™ Soluble Dietary Fiber; BAHEAL Medical Inc., Qingdao, China and Fengning Ping'an High-tech Industrial Co., Ltd., Heber, China). Because of the differential sex phenotype following different maternal nutrition, this project researched only male offspring. At weaning, male offspring (*n* = 8 per group) were sacrificed. Mice were fasted for 10 h and anesthetized with chloral hydrate, and a blood sample was collected from the intraorbital retrobulbar plexus. The cecal contents were quickly removed, snap frozen on dry ice, and then stored at −80°C for further analysis. Figure 1 shows the experimental protocol.

### Measurement of Body Weight and Fasting Blood Glucose

The body weight of both the mother and pups was measured. Blood was collected from a tail bleed and analyzed to test glucose levels using a Contour TS glucometer (Bayer, Hamburg, Germany).

### Oral Glucose Tolerance Test (OGTT)

At weaning, an OGTT was performed to assess glucose tolerance in pups. After 10 h of fasting, pups were given a glucose load (2.0 g/kg body weight) by gavage. Before (0 min) and at 30, 60, and 120 min after the gavage, the blood glucose levels were measured. The area under the glucose tolerance curve (AUC) of the OGTT was calculated as previously described (19).

### Serum Insulin Assay and Homeostasis Model Assessment of Insulin Resistance (HOMA-IR)

At weaning, pups were fasted for 10 h to measure serum insulin by using an ELISA kit (Millipore, Billerica, MA, USA). Insulin sensitivity was assessed using HOMA-IR as previously described (19).

### Microbial Sampling and DNA Isolation

Fifty micrograms of cecal contents was used for metagenomic DNA isolation using a TIANamp Stool DNA Kit (TIANGEN, Beijing, China). The quality and quantity of DNA was assessed using agarose gel electrophoresis and fluorometry (Qubit® dsDNA Assay Kit, Life Technologies, CA, USA).

### Metagenomic Library Construction and Metagenomic Sequencing

One microgram of DNA was sheared to 350 bp fragments by sonication. After polishing and ligation with a full-length adaptor, DNA fragments were amplified using an NEBNext® Ultra™ DNA Library Prep Kit from Illumina (New England BioLabs Inc, Ipswich, MA, USA). Then, PCR amplification products were purified (AMPure XP System, Beckman Coulter, Woerden, Netherlands). DNA libraries underwent size distribution via an Agilent 2100 Bioanalyzer. Finally, DNA libraries were sequenced on the Illumina HiSeq 2000 Platform (Beijing Compass Biotechnology Company, China). Paired-end reads were generated for further processing.

### Metagenome Preprocessing and Assembly

First, we removed the adaptors and low quality reads. Then, reads were filtered to exclude the host DNA genome based on the *Mus musculus* reference genome using Bowtie 2.2.4 software (20). Then, the clean data were assembled into contigs and analyzed by SOAPdenovo software v2.04 (21).

### Gene Prediction, Taxonomy, and Functional Profiling

After assembling contigs, open reading frames (ORFs) were predicted by MetaGeneMark software (22). DIAMOND software was used to blast the Unigenes to the sequences of bacteria that were extracted from NCBI (23). For beta diversity, principal component analysis (PCA) plots were constructed. LEfSe analysis was conducted by using LEfSe software (24). DIAMOND software was used to blast the Unigenes to a KEGG functional database (25).

### Statistics

The data are expressed as the mean ± SD. Differences among the groups were analyzed using one-way ANOVA followed by Tukey's *post hoc* test. For sequencing data, statistical analyses were performed using R software (v. 2.15.3). For determination of the abundance of genes, taxonomies, and KEGG ontologies, the Mann-Whitney test was used for statistics between two groups, and the Kruskal-Wallis test was used for comparisons among three groups. Statistical analyses were performed in GraphPad Prism 6 (GraphPad Software Inc., CA, USA). Statistical significance was defined as *P* < 0.05.

## Results

### Maternal Body Weight and Changes in Fasting Blood Glucose

**Figure 2:**
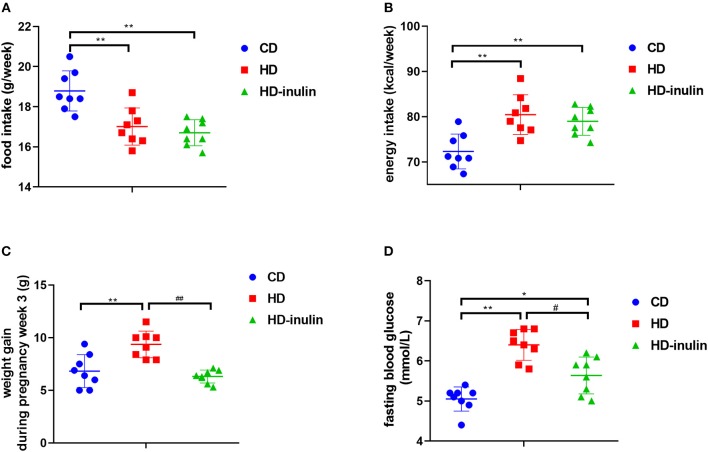
Maternal food intake, energy intake, body weight and fasting blood glucose in different groups at weaning time. **(A)** Maternal weekly food intake during gestation and lactation period. **(B)** Maternal weekly energy intake during gestation and lactation period. **(C)** Maternal body weight. **(D)** Maternal fasting blood glucose. The data were shown as mean ± SD. *n* = 8 in each group. *P < 0.05, **P < 0.01 vs. CD group; ^#^*P* < 0.05, ^*##*^*P* < 0.01 vs. HD group. CD, control diet; HD, high fat diet; HD-inulin, high fat diet with inulin supplement.

Before consuming a HD, dam body weight between groups did not differ (*P* > 0.05). After eating a HD for 4 weeks, the HD group had a higher body weight than the CD group (19.77 ± 1.46 g vs. 17.28 ± 1.16 g, *P* < 0.01). Despite decreased food intake in the HD group compared with the CD group (Figure 2A, *P* < 0.01), the energy intake of the HD group was higher than that of the CD group during the pregnancy period (Figure 2B, *P* < 0.01). Accordingly, the body weight change in HD mice was 139% of that in CD mice (Figure 2C, *P* < 0.01). Although no significant difference in food intake and energy intake was observed between the HD and HD-inulin groups, the body weight change in HD-inulin dams was 33.9% lower than that in the HD mice during the pregnancy period (Figure 2C, *P* < 0.01). Similarly, maternal HD feeding increased fasting blood glucose by 24.8% compared with CD mice (Figure 2D, *P* < 0.01). Interestingly, fasting blood glucose was reduced by 11.3% in HD-inulin dams compared with HD mice (Figure 2B, *P* < 0.05).

### Offspring Body Weight

**Figure 3:**
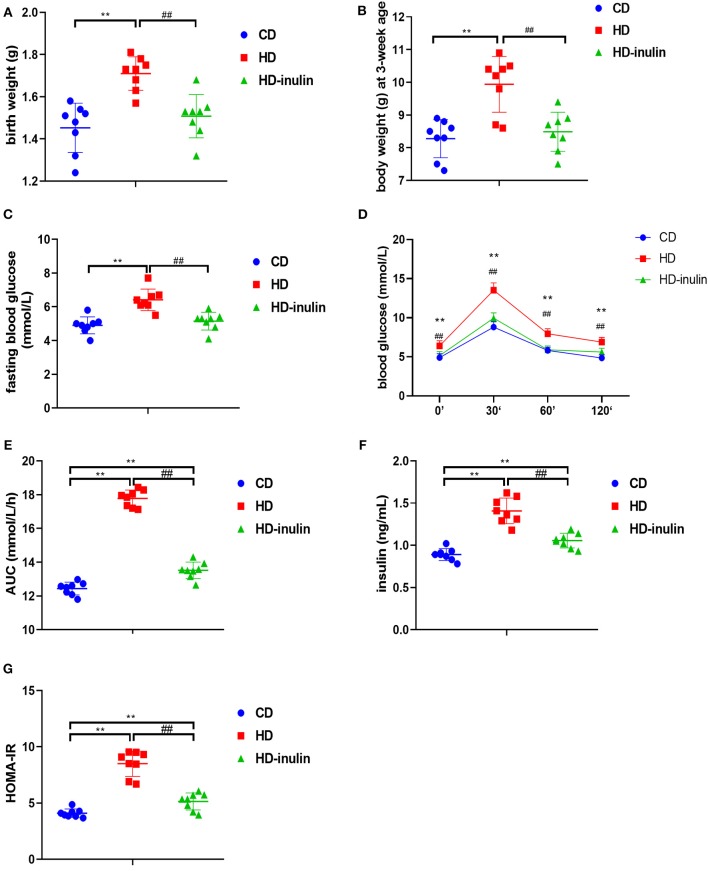
Metabolic variables of male mice offspring in different groups. **(A)** birth weight; **(B)** body weight at weaning; **(C)** fasting blood glucose; **(D)** oral glucose tolerance test (OGTT); **(E)** area under curve (AUC) in OGTT; **(F)** serum insulin; **(G)** HOMA-IR. The data were shown as mean ± SD. *n* = 8 in each group. **P < 0.01 vs. CD group; ^*##*^*P* < 0.01 vs. HD group. CD, control diet; HD, high fat diet; HD-inulin, high fat diet with inulin supplement.

Litters from HD dams weighed more than litters from CD dams both at birth and at weaning time (*P* < 0.01, Figures 3A,B). However, the pups in the HD-inulin group had reduced body weight compared with pups in the HD group both at birth and at weaning (*P* < 0.01, Figures 3A,B).

### Offspring Glucose Metabolic Index, Serum Insulin, and HOMA-IR

Compared with pups from CD dams, pups from dams fed a HD displayed increased fasting blood glucose, blood glucose during OGTT, and AUC during OGTT (*P* < 0.01, Figures 3C–E). Offspring from HD-inulin dams had lower fasting blood glucose, and blood glucose and AUC during OGTT (*P* < 0.01, Figures 3C–E). Fasting insulin and HOMA-IR were significantly higher in pups from HD dams than in pups from CD controls (*P* < 0.01, Figures 3F,G). Maternal inulin supplementation reduced serum fasting insulin and HOMA-IR in male pups at weaning (*P* < 0.01, Figures 3F,G).

### Characterization of Gut Microbiota

Cecal contents from 15 mice (*n* = 5 per group) were used to perform whole-metagenome shotgun sequencing to understand the gut microbial composition. After quality control, we acquired a total of 113.5 Gbp of high-quality metagenomic data (7.56 ± 0.62 Gbp per sample) for further analysis. The sequence data generated in this study were submitted to the NCBI Sequence Read Archive database (accession number PRJNA552163). After *de novo* assembly and gene data calling, we constructed a non-redundant gene catalog of all cecal contents containing 1,048,576 genes. This gene catalog was qualified for further gut microbial analysis.

### Gut Microbial Beta Diversity

**Figure 4:**
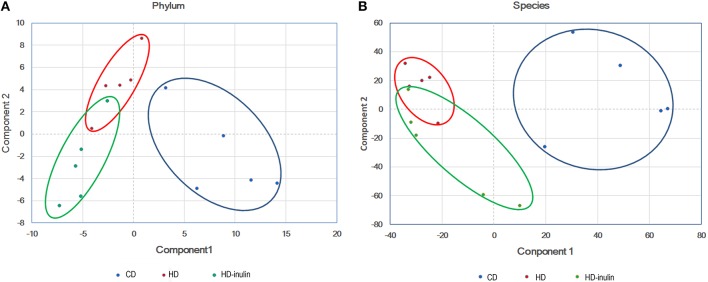
Principal component analysis plot at phylum level **(A)** and species level **(B)**. CD, control diet; HD, high fat diet; HD-inulin, high fat diet with inulin supplement.

According to the principal component analysis of the bacterial abundance, the gut microbial communities were significantly different among the three groups (Figure 4). Offspring gut microbial composition was affected by both maternal HD and maternal inulin supplementation.

### Relative Abundance of Gut Microbiota at the Phyla Level

**Figure 5:**
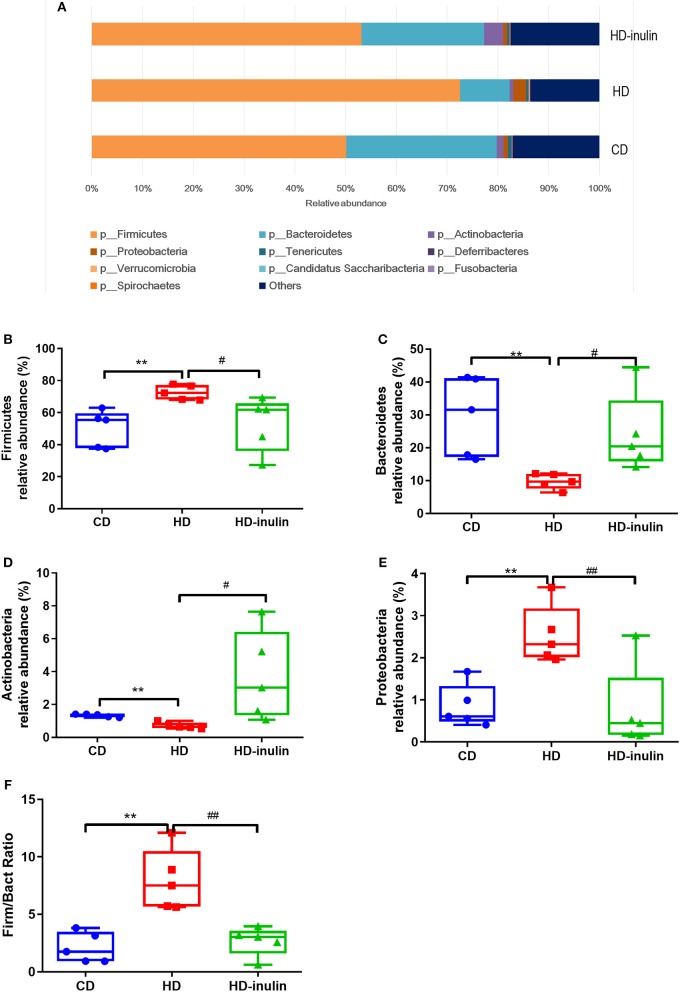
The relative abundance of bacterial population at phylum level **(A)**. The abundance of Firmicutes **(B)**, Bacteroidetes **(C)**, Actinobacteria **(D)**, Proteobacteria **(E)**, and Firmicutes/Bacteroidetes (Firm/Bact) ratios **(F)** in pups from CD, HD, and HD-inulin dams. Kruskal-Wallis non-parametric test, followed by the Wilcoxon tests. The data were shown as median (min-max). *n* = 5 in each group. **P < 0.01 vs. CD group; ^#^*P* < 0.05, ^*##*^*P* < 0.01 vs. HD group. CD, control diet; HD, high fat diet; HD-inulin, high fat diet with inulin supplement.

The distribution and relative abundance of bacterial taxa at the phyla level are shown in Figures 5A–F. Offspring gut microbiota were distributed across 10 bacterial phyla, including *Firmicutes, Bacteroidetes, Actinobacteria, Proteobacteria, Tenericutes, Deferribacteres, Verrucomicrobia, Candidatus Saccharibacteria, Fusobacteria, and Spirochaetes*. The relative abundance analysis revealed a significant increase in the abundance of *Firmicutes* and *Proteobacteria* in pups from HD dams (*P* < 0.05, Figures 5B,D). On the other hand, the abundance of *Bacterodetes* and *Actinobacteria* was reduced in pups from HD dams (*P* < 0.05, Figures 5C,E). Inulin supplementation moderated the disturbance of the abundance of *Firmicutes, Actinobacteria, Bacteroidetes*, and *Proteobacteria* (*P* < 0.05, Figures 5B–E). The ratio of *Firmicutes* and *Bacteroidetes* in pups from HD dams was greatly increased (*P* < 0.05, Figure 5F). Inulin supplementation moderated this ratio (*P* < 0.05, Figure 5F).

### LEfSe Analysis in Different Groups at the Species Level

**Figure 6:**
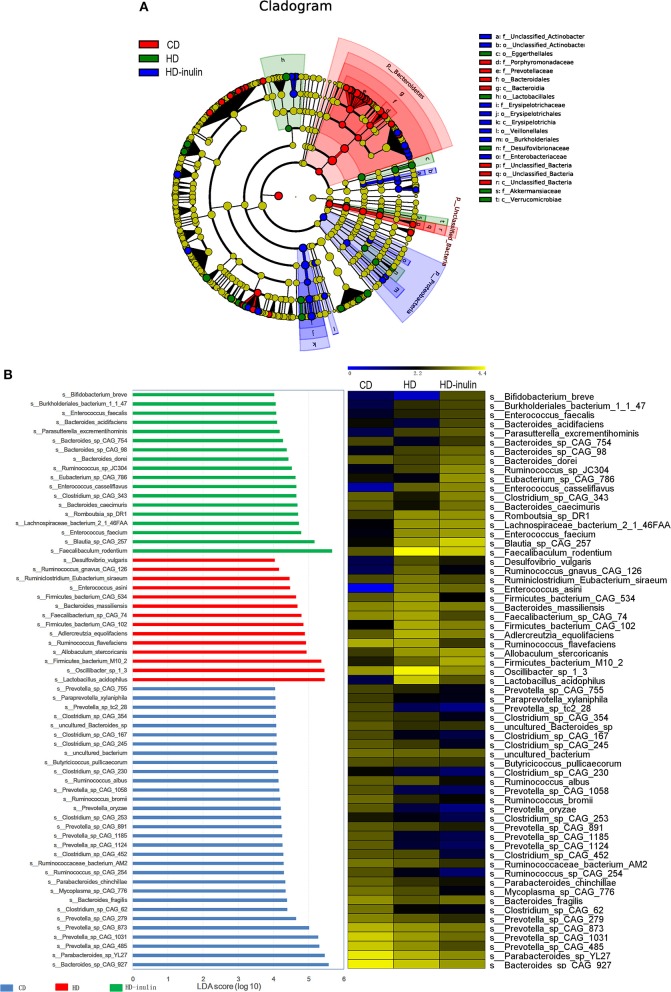
The LEfSe analysis of the gut microbiota in pups from HD-inulin dams differed significantly from that in pups from CD and HD dams. **(A)** Differences are represented in the color of the most abundant group. **(B)** The left histogram shows the lineages with LDA values of 4.0 or higher as determined by LEfSe in species level. The right heap map shows the relative abundance (log_10_ transformation). CD, control diet; HD, high fat diet; HD-inulin, high fat diet with inulin supplement.

**Figure 7:**
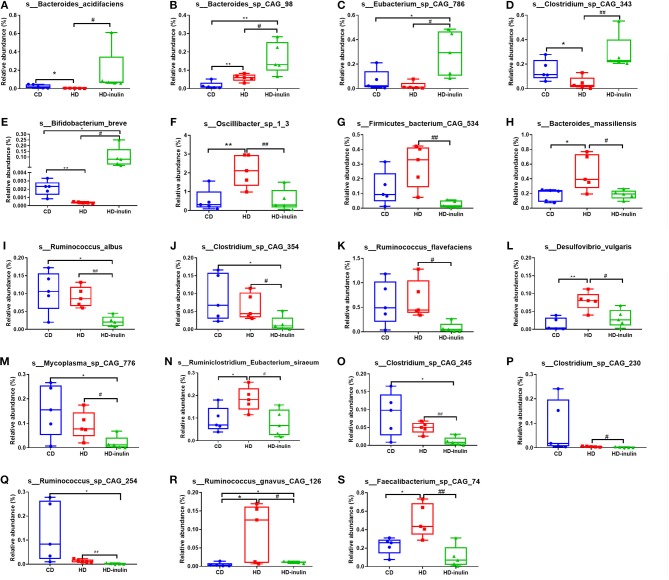
The relative abundance of bacterial population at species level. *Bacteroides_acidifaciens* **(A)**, *Bacteroides_sp_CAG_98* **(B)**, *Eubacterium_sp_CAG_786* **(C)**, *Clostridium_sp_CAG_343* **(D)**, and *Bifidobacterium_breve* **(E)**, *Oscillibacter_sp_1_3* **(F)**, *Firmicutes_bacterium_CAG_534* **(G)**, *Bacteroides_massiliensis* **(H)**, *Ruminococcus_albus* **(I)**, *Clostridium_sp_CAG_354* **(J)**, *Ruminococcus_flavefaciens* **(K)**, *Desulfovibrio_vulgaris* **(L)**, *Mycoplasma_sp_CAG_776* **(M)**, *Ruminiclostridium_Eubacterium_siraeum* **(N)**, *Clostridium_sp_CAG_245* **(O)**, *Clostridium_sp_CAG_230* **(P)**, *Ruminococcus_sp_CAG_254* **(Q)**, *Ruminococcus_gnavus_CAG_126* **(R)**, and *Faecalibacterium_sp_CAG_74* **(S)** in pups from CD, HD, and HD-inulin dams. Kruskal-Wallis non-parametric test, followed by the Wilcoxon tests. The data were shown as median (min–max). *n* = 5 in each group. *P < 0.05, **P < 0.01 vs. CD group; ^#^*P* < 0.05, ^*##*^*P* < 0.01 vs. HD group. CD, control diet; HD, high fat diet; HD-inulin, high fat diet with inulin supplement.

We further compared changes in the gut microbiota among the three groups by using LEfSe analysis. The histogram of the LDA scores further revealed a clear difference among pups from the CD, HD, and HD-inulin dams at the species level (Figure 6). Sixty-three bacterial species changed significantly among the pups from the CD, HD, and HD-inulin dams. Among these bacteria species, 19 species showed a significant difference between the pups from HD-inulin dams and HD dams (*P* < 0.05). *Bacteroides_acidifaciens* (*P* < 0.05), *Bacteroides_sp_CAG_98* (*P* < 0.05), *Eubacterium_sp_CAG_786* (*P* < 0.05), *Clostridium_sp_CAG_343* (*P* < 0.01), and *Bifidobacterium_breve* (*P* < 0.05) were significantly elevated in pups from HD-inulin dams vs. pups from HD dams. However, *Oscillibacter_sp_1_3* (*P* < 0.01), *Firmicutes_bacterium_CAG_534* (*P* < 0.01), *Bacteroides_massiliensis* (*P* < 0.05), *Ruminococcus_albus* (*P* < 0.05), *Clostridium_sp_CAG_354* (*P* < 0.05), *Ruminococcus_flavefaciens* (*P* < 0.05), *Desulfovibrio_vulgaris* (*P* < 0.05), *Mycoplasma_sp_CAG_776* (*P* < 0.05), *Ruminiclostridium_Eubacterium_siraeum* (*P* < 0.05), *Clostridium_sp_CAG_245* (*P* < 0.01), *Clostridium_sp_CAG_230* (*P* < 0.05), *Ruminococcus_sp_CAG_254* (*P* < 0.01), *Ruminococcus_gnavus_CAG_126* (*P* < 0.05), and *Faecalibacterium_sp_CAG_74* (*P* < 0.01) were reduced significantly in pups from HD-inulin dams vs. HD dams (Figure 7).

### KEGG Metabolic Pathways

We used the KEGG pathway database to analyze functional metagenomic profiles. The comparison among groups indicated significant differences in 38 metabolic functions (*P* < 0.05, Table 1). Most of these features revealed a similar abundance between pups from CD and HD-inulin dams. A series of glucose and lipid metabolism pathways were modified in pups from HD-inulin dams compared with pups from HD dams, such as glycolysis/gluconeogenesis, citrate cycle (TCA cycle), fatty acid biosynthesis, adipocytokine signaling pathway, glucagon signaling pathway, type II diabetes mellitus, and carbohydrate digestion and absorption.

**Table 1:** The relative abundance (%) of bacterial groups that showed statistical significance of KEGG pathway categories among different groups.

**KEGG function level 3**	**CD**	**HD**	**HD-inulin**	***P*-value**	
				**HD vs. CD**	**HD-inulin vs. HD**
Glycolysis/Gluconeogenesis	0.6148(0.5570–0.6355)	0.7049(0.6043–0.7745)*	0.57650(0.53656–0.66263)^#^	0.03260	0.04934
Citrate cycle (TCA cycle)	0.2828(0.2680–0.3155)	0.2597(0.2323–0.2822)*	0.31907(0.25165–0.36493)^#^	0.03499	0.03844
Fatty acid biosynthesis	0.2342(0.2135–0.2573)	0.1920(0.1875–0.2256)*	0.22575(0.20333–0.24197)^#^	0.01960	0.04073
Geraniol degradation	0.0001(0.0000–0.0002)	0.0010(0.0004–0.0025)*	0.00011(0.00007–0.00038)^#^	0.03166	0.03742
Lysine biosynthesis	0.2983(0.2841–0.3296)	0.3294(0.3021–0.3332)*	0.28247(0.25319–0.33476)^#^	0.04331	0.04813
Lysine degradation	0.0304(0.0257–0.0359)	0.0481(0.0466–0.0529)**	0.03409(0.02443–0.04769)^#^	0.00179	0.03139
Furfural degradation	0.0000(0.0000–0.0001)	0.0001(0.0001–0.0001)*	0.00000(0.00000–0.00005)^#^	0.01573	0.03019
Tryptophan metabolism	0.0218(0.0162–0.0222)	0.0353(0.0310–0.0393)**	0.02619(0.01823–0.03353)^#^	0.00390	0.04139
Phenylalanine, tyrosine and tryptophan biosynthesis	0.3522(0.3181–0.3744)	0.3180(0.2895–0.3444)*	0.34770(0.32533–0.39867)^#^	0.03330	0.02792
D-Alanine metabolism	0.0517(0.0479–0.0564)	0.0612(0.0482–0.0720)*	0.04862(0.04265–0.05741)^#^	0.04520	0.03154
Other glycan degradation	0.1488(0.1202–0.2094)	0.1025(0.0970–0.1262)*	0.17478(0.11970–0.26930)^#^	0.03166	0.03805
Amino sugar and nucleotide sugar metabolism	0.7181(0.6680–0.7482)	0.8406(0.7493–0.8665)*	0.67503(0.60146–0.80699)^#^	0.01718	0.03934
Peptidoglycan biosynthesis	0.4221(0.4010–0.4480)	0.4537(0.4386–0.4615)*	0.36959(0.34915–0.45002)^#^	0.02066	0.02034
Sphingolipid metabolism	0.1130(0.1059–0.1476)	0.0937(0.0879–0.1058)*	0.12882(0.09625–0.17644)^#^	0.03166	0.02805
Pyruvate metabolism	0.4972(0.4639–0.5164)	0.5507(0.4991–0.5585)*	0.45720(0.43866–0.54814)^#^	0.01683	0.02934
Toluene degradation	0.0002(0.0001–0.0003)	0.0015(0.0002–0.0022)*	0.00049(0.00018–0.00081)*^#^	0.01625	0.04934
Vitamin B6 metabolism	0.0891(0.0814–0.0979)	0.0756(0.0667–0.0801)*	0.08564(0.07998–0.10059)^#^	0.01620	0.02803
Folate biosynthesis	0.1733(0.1254–0.1937)	0.1198(0.0987–0.1432)*	0.00158(0.00130–0.00181)^#^	0.03425	0.03128
Limonene and pinene degradation	0.0074(0.0057–0.0111)	0.0148(0.0119–0.0189)*	0.00005(0.00002–0.00014)^#^	0.01300	0.00934
Caprolactam degradation	0.0019(0.0002–0.0074)	0.0028(0.0026–0.0029)	0.00002(0.00001–0.00002)^#^	0.30751	0.02752
Flavone and flavonol biosynthesis	0.0046(0.0028–0.0064)	0.0025(0.0019–0.0035)	0.00004(0.00003–0.00004)^#^	0.04153	0.03792
ABC transporters	0.9351(0.7441–1.5006)	1.5091(1.4626–1.6103)*	0.00187(0.00101–0.01708)^#^	0.01960	0.04584
Ribosome biogenesis in eukaryotes	0.0193(0.0176–0.0201)	0.0246(0.0237–0.0252)**	0.00020(0.00016–0.00025)^#^	0.00159	0.03139
Ribosome	1.1228(1.0636–1.3193)	1.3400(1.3128–1.3650)*	0.01147(0.01099–0.01296)^#^	0.02666	0.00302
RNA polymerase	0.1164(0.0976–0.1221)	0.1253(0.1240–0.1342)*	0.00105(0.00099–0.00122)^#^	0.02489	0.00317
Bacterial secretion system	0.3061(0.2976–0.3245)	0.3300(0.3252–0.3336)*	0.00307(0.00237–0.00321)^#^	0.01300	0.03128
PPAR signaling pathway	0.0591(0.0479–0.0702)	0.0476(0.0419–0.0549)*	0.00066(0.00046–0.00077)^#^	0.04254	0.04936
Base excision repair	0.2119(0.2023–0.2278)	0.2394(0.2347–0.2505)*	0.21334(0.18743–0.23365)^#^	0.01048	0.00726
HIF-1 signaling pathway	0.0494(0.0434–0.0553)	0.0615(0.0520–0.0642)*	0.05271(0.05079–0.05575)^#^	0.01960	0.01655
Sulfur relay system	0.0934(0.0889–0.1164)	0.1249(0.1200–0.1327)*	0.08055(0.01371–0.13565)^#^	0.01145	0.03893
Apoptosis	0.0149(0.0068–0.0158)	0.0030(0.0013–0.0043)*	0.01686(0.00387–0.05312)^#^	0.01300	0.04234
Adipocytokine signaling pathway	0.0346(0.0337–0.0574)	0.0266(0.0194–0.0329)*	0.03450(0.02599–0.05028)^#^	0.02666	0.04397
Glucagon signaling pathway	0.0892(0.0816–0.0971)	0.1270(0.1009–0.1336)*	0.70773(0.09824–0.73901)*^#^	0.01573	0.04893
Type II diabetes mellitus	0.0226(0.0222–0.0239)	0.0264(0.0258–0.0274)**	0.02215(0.02147–0.02677)^#^	0.00381	0.02674
Carbohydrate digestion and absorption	0.0155(0.0097–0.0323)	0.0071(0.0052–0.0132)*	0.03536(0.00509–0.04880)^#^	0.04024	0.02193
Protein digestion and absorption	0.0160(0.0069–0.0175)	0.0042(0.0016–0.0058)*	0.02259(0.00643–0.06965)^#^	0.01573	0.04139
Proteoglycans in cancer	0.0191(0.0175–0.0199)	0.0245(0.0236–0.0247)**	0.02156(0.01852–0.02434)*^#^	0.00119	0.01655
Renal cell carcinoma	0.0030(0.0022–0.0047)	0.0074(0.0048–0.0083)	0.00434(0.00282–0.00604)^#^	0.01620	0.01655

CD, control diet; HD, high fat diet; HD-inulin, high fat diet with inulin supplement.

* *P < 0.05*,

** P < 0.01 vs. CD group;

# *P < 0.05, ^##^P < 0.01 vs. HD group*.

## Discussion

In this study, female mice fed a HD exhibited higher energy intake and weight gain during the pregnancy period. In spite of the change in energy intake, inulin treatment reduced weight gain during pregnancy and fasting blood glucose in female mice. In rodent experiments, several groups found that inulin-type fructans dramatically reduced epididymal, inguinal, and visceral adipose tissue fat masses by 30–40% (26–30). Previous studies showed that inulin-type fructans effectively reduced fasting blood glucose in both T2D patients (31) and diabetic rats (15, 17, 32). Meta-analysis also revealed that dietary prebiotic consumption improved satiety and reduced postprandial blood glucose and insulin levels (33).

Maternal HD feeding led to an increase in blood glucose and insulin resistance in male offspring at weaning. This result is consistent with previous literature (34–36). Interestingly, our results showed that early maternal inulin intervention reduces fasting blood glucose and moderates glucose intolerance and insulin resistance in offspring from HD dams. Other studies also prove that prebiotics can reduce glycemia in offspring in rats (37–39).

The gut microbiome plays an important role in the pathogenesis of various health disorders, such as diabetes (40) and obesity (41). In the current study, the β-diversity of the gut microbiota showed a significant difference among offspring from CD, HD, and HD-inulin dams.

Our results showed that offspring from HD-inulin dams exhibited marked changes in microbial abundance at the phylum level compared with offspring from HD dams. First, the ratio of *Firmicutes* to *Bacteroidetes* in pups from HD dams was greatly increased, and inulin supplementation moderated this ratio. Previous studies have shown that the abundance of *Firmicutes* is associated with metabolic disease in mice (9, 42). Second, offspring from HD dams had a lower *Actinobacteria* abundance than those from CD dams. Maternal inulin supplementation moderated *Actinobacteria* abundance. Although *Actinobacteria* make up a tiny proportion, they are critical for the maintenance of gut homeostasis (43). As an important probiotic, *Bifidobacterium*, all under the classes of *Actinobacteria*, has favorable effects on human health (43). Type 1 diabetic children have a lower abundance of *Actinobacteria* compared with healthy children (44). Moreover, there was a significant increase in the abundance of *Proteobacteria* in offspring from HD dams compared to CD dams. A previous study showed that dietary inulin affects the gut microbiota of healthy suckling piglets fed a normal diet (45). In a rodent study, prebiotic intervention may change gut microbiota richness and diversity in mice fed either a normal diet and or a high-fat diet (45, 46). In our research, maternal inulin supplementation reduced the abundance of *Proteobacteria*. An increased abundance of *Proteobacteria* is an indicator of an unhealthy gut microbial community (47). Type 2 diabetic mice and humans have high levels of *Proteobacteria* (48, 49). Collectively, our study confirms that maternal inulin supplementation moderates dysbiosis in offspring of HD dams.

At the species level, the abundance of *Bifidobacterium_breve* was decreased in offspring of HD dams compared to those of CD dams. HD-inulin group pups had a higher *Bifidobacterium_breve* relative abundance*. Bifidobacterium* is one of the most abundant probiotic species present in the mammalian gut (50). Previous studies indicate that a lower abundance of *Bifidobacterium* is a hallmark of a type 2 diabetic gut microbiota and accelerates the progression of type 2 diabetes (51, 52). Type 2 diabetic, overweight and obese patients have a lower abundance of *Bifidobacterium* than healthy adults and children (53–55). *Bifidobacterium* modulates glucose metabolism, improves insulin resistance, and gut barrier function, and stimulates the host immune system (56, 57). Paul et al. found that oligofructose treatment during gestation increased the maternal relative abundance of *Bifidobacterium* compared to rats fed a high-fat/sucrose diet (37). Interestingly, the increased abundance of *Bifidobacterium* is similar between mothers and pups. According to previous literature (58), the increased abundance of *Bifidobacterium* in offspring affected by maternal inulin supplementation may also be linked to the observed improvement in HOMA-IR scores. Some data raised the role of gut microbiota in metabolic programming (59). Gut microbiota is one of the most important environmental factors that will affect offspring metabolism (60). Although we cannot exclude the transmission of microbial components from mothers to pups during vaginal delivery and breastfeeding (61), this study could provide some evidence that inulin intervention during gestation could increase maternal *Bifidobacterium* abundance, and may have colonized the pup gut.

Our findings indicate that maternal inulin supplementation induced a marked increase in the abundance of *Bacteroides_acidifaciens, Eubacterium_sp_CAG_786*, and *Clostridium_sp_CAG_343* in offspring. *Bacteroides, Eubacterium*, and *Clostridium* are butyrate-producing bacteria (62, 63). Butyrate promotes intestinal barrier function to maintain colonic health through increased fatty acid oxidation (64, 65). Type 2 diabetic patients have reduced levels of butyrate-producing bacteria (66). A previous study showed that *Bacteroides_acidifaciens* improves insulin sensitivity in mice (67). Oral administration of *Eubacterium* improved insulin sensitivity in db/db mice (68). *Clostridium* improved fasting glucose, glucose tolerance and insulin tolerance in STZ induced HD-fed diabetic C57BL/6J mice (69). Butyrate enhances the secretion of the gut satiety hormones glucagon-like peptide-1 (GLP-1) and peptide YY (PYY) (70). GLP-1 and PYY played an important role in body weight reduction. A previous study showed that maternal oligofructose treatment increased plasma GLP-1 and PYY levels both in rat mothers and pups (37). Therefore, another explanation for the effect of maternal inulin treatment on glucose metabolism in pups might be the transfer of gut satiety hormones from mothers to pups through the placenta.

*Ruminococcus* is considered a butyrate-producing acid bacteria (71). However, in this study, the relative abundance of several *Ruminococcus* was lower in the HD-inulin group than in the HD group, such as *Ruminococcus_albus, Ruminococcus_flavefaciens, Ruminococcus_sp_CAG_254*, and *Ruminococcus_gnavus_CAG_126*. Researchers found that *Ruminococcus* was highly abundant in adults with T2DM (72). Random blood glucose was positively correlated with *Ruminococcus* in rats (73). A number of *Ruminococcus* species are thought to be related to metabolic diseases. *Ruminococcus* also acts as transient blooms of proinflammation (74). *Ruminococcus_gnavus* has been found to secrete a unique L-rhamnose oligosaccharide that induces tumor necrosis factor alpha (TNFa), a major pro-inflammatory cytokine (75).

The abundance of *Oscillibacter_sp_1_3* and *Bacteroides_massiliensis* in offspring from HD-inulin dams was significantly reduced compared to HD dams. *Bacteroides_massiliensis* was significantly enriched in obese children (76) and in German adults who were unsuccessful with weight loss (77). It is also positively correlated with body mass index (BMI) z-scores (76). *Oscillibacter* was strongly associated with the diabetic phenotype (78) and also positively correlated with gut permeability (79).

Metagenomic function prediction is based on the relative expression abundance of each bacterial taxonomy, which belongs to each metabolic pathway. KEGG pathway analysis seems to show differences in 38 metabolic functions among offspring from CD, HD, and HD-inulin dams. Specifically, maternal inulin supplementation may alter gut microbiota involved in glucose and lipid metabolism, such as glycolysis/gluconeogenesis, citrate cycle (TCA cycle), fatty acid biosynthesis, adipocytokine signaling pathway, glucagon signaling pathway, type II diabetes mellitus, and carbohydrate digestion and absorption. Previous studies indicated the beneficial effect of prebiotics on metabolic disorders, such as obesity and type 2 diabetes (80, 81). Given that metagenomic function prediction is based on the relative expression abundance of gut microbiota, more experiments about the metabolic function of gut microbiota need to be performed.

## Conclusion

In conclusion, maternal inulin supplementation has beneficial effects on glucose metabolism in offspring, including improvements in glucose intolerance and insulin resistance. Importantly, maternal inulin supplementation increased the abundance of *Bifidobacterium* and several butyrate-producing bacteria. Thus, maternal inulin supplementation is promising for the prevention of metabolic disorders in the offspring.

## Data Availability Statement

The datasets analyzed in this manuscript are not publicly available. Requests to access the datasets should be directed to The datasets supporting the conclusions of this manuscript are available from the corresponding author (xiaoxh2014@vip.163.com) on reasonable request.

## Ethics Statement

All animal experimental protocols were approved by the Animal Care Committee of Peking Union Medical Hospital (Permit Number: MC-07-6004).

## Author Contributions

XX conceived and designed the experiments. QZ, JZ, TW, and XW performed the experiments. MY, ML, and FP analyzed the data. XX contributed reagents, materials, and analysis tools. QZ wrote the paper.

### Conflict of Interest

The authors declare that the research was conducted in the absence of any commercial or financial relationships that could be construed as a potential conflict of interest.
